# Effect of MiR‐100‐5p on proliferation and apoptosis of goat endometrial stromal cell in vitro and embryo implantation in vivo

**DOI:** 10.1111/jcmm.17226

**Published:** 2022-04-12

**Authors:** Li Ma, Meng Zhang, Fangjun Cao, Jincheng Han, Peng Han, Yeting Wu, Renyi Deng, Guanghui Zhang, Xiaopeng An, Lei Zhang, Yuxuan Song, Binyun Cao

**Affiliations:** ^1^ 12469 College of Animal Science and Technology Northwest A&F University Yangling China; ^2^ Shaanxi University of Chinese Medicine Xianyang China; ^3^ Shaanxi Institute of Zoology Xi’an China; ^4^ 12469 College of Food Science and Engineering Northwest A&F University Yangling China; ^5^ 12469 Department of Foreign Languages Northwest A&F University Yangling China; ^6^ 12469 College of Innovation and Experiment Northwest A&F University Yangling China

**Keywords:** Circ‐9110, dairy goat, endometrial stromal cells, *HOXA1*, miR‐100‐5p

## Abstract

The growth of endometrial stromal cells (ESCs) at implantation sites may be a potential factor affecting the success rate of embryo implantation. Incremental proofs demonstrated that ncRNAs (e.g. miRNAs, lncRNAs and circRNAs) were involved in various biological procedures, including proliferation and apoptosis. In this study, the role of miR‐100‐5p on proliferation and apoptosis of goat ESCs in vitro and embryo implantation in vivo was determined. The mRNA expression of miR‐100‐5p was significantly inhibited in the receptive phase (RE) rather than in the pre‐receptive phase (PE). Overexpression of miR‐100‐5p suppressed ESCs proliferation and induced apoptosis. The molecular target of MiR‐100‐5p, *HOXA1*, was confirmed by 3′‐UTR assays. Meanwhile, the product of *HOXA1* mRNA RT‐PCR increased in the RE more than that in the PE. The *HOXA1*‐siRNA exerted significant negative effects on growth arrest. Instead, incubation of ESCs with miR‐100‐5p inhibitor or overexpressed *HOXA1* promoted the cell proliferation. In addition, Circ‐9110 which acted as a sponge for miR‐100‐5p reversed the relevant biological effects of miR‐100‐5p. The intrinsic apoptosis pathway was suppressed in ESCs, revealing a crosstalk between Circ‐9110/miR‐100‐5p/*HOXA1* axis, PI3K/AKT/mTOR, and ERK1/2 pathways. To further evaluate the progress in study on embryo implantation regulating mechanism of miR‐100‐5p in vivo, the pinopodes of two phases were observed and analysed, suggesting that, as similar as in situ, miR‐100‐5p was involved in significantly regulating embryo implantation in vivo. Mechanistically, miR‐100‐5p performed its embryo implantation function through regulation of PI3K/AKT/mTOR and ERK1/2 pathways by targeting Circ‐9110/miR‐100‐5p/*HOXA1* axis in vivo.

## 1 INTRODUCTION

Successful breeding in mammals requires precise timing control and complex interaction in the uterine endometrium.^1^ Abnormal endometrial function results in female infertility by causing implantation failure, recurrent pregnancy loss and other pathologies.^1^ A receptive endometrium is the key to successful implantation, which depends on stromal cell proliferation and differentiation.^2^ The morphology and function of a receptive endometrium changes during its development because of orchestrated interactions among many processes, including cell proliferation and apoptosis.^3^, ^4^ Moreover, it is noteworthy that complex biological processes and pathways may involve in endometrial receptivity.^5^ Significantly, the proliferation and apoptosis of endometrial cells and related regulatory mechanisms exert important effect on the successful implantation of dairy goat embryos. The microRNAs (miRNAs), which are small endogenously conserved non‐coding regulatory RNA molecules, recently have been identified to be a promising anticancer candidate for the treatment of cancers. However, underlying molecular mechanisms of many vital microRNAs in endometrial receptivity remain elusive.

The microRNAs which silenced their target genes at the post‐transcriptional level regulated many signalling pathway in varied of cells.^6^ Besides, some exceptional miRNAs changed drastically at the different developmental stages of cells development.^7^ In recent years, many investigations have indicated that miRNAs are involved in cells growth, cells differentiation and programmed cells death. MicroRNAs acted as gatekeepers of apoptosis.^8^ For example, microRNA‐183 promoted proliferation and invasion in oesophageal squamous cell carcinoma by targeting programmed cell death.^9^ Our group primarily conduct research to study the different effects of different microRNA in dairy goats breeding. The related research work is as follows: Mir‐101‐3p exhibited differential expression level in the ovaries of single lamb and multi lamb dairy goats, indicating that mir‐101‐3p played an important role in regulating the growth and development of follicles.^10^ In addition, novel‐mir‐3880 played a regulatory role in breast development and milk synthesis, which showed high expression in the mammary gland of dairy goats at the peak of lactation.^11^ What is more, Circ‐8073, as a ceRNA, cooperated with mir‐449/34 family through CEP55‐FOXM1‐VEGFA to regulate the establishment of milk goat receptive endometrium.^12^ Based on their special regulatory function in cells, in dairy goat breeding, some miRNAs play important roles in the establishment of endometrial receptivity. MiR‐449a regulated caprine endometrial stromal cell apoptosis and endometrial receptivity.^13^ Testin was regulated by circRNA3175‐miR182 and inhibited endometrial epithelial cell apoptosis in pre‐receptive endometrium of dairy goats.^11^ In our previous study, the RNA‐seq analysis of dairy goats endometria with two stages demonstrated that few miRNAs were downregulated significantly in the RE but not in the PE.^14^ The miRNA profile related to the biology of the goat receptive endometrium during embryo implantation was constructed, suggesting that miRNAs might play important roles in the formation of endometrial receptivity. There were 110 up‐expressed miRNAs and 33 down‐expressed miRNAs in the RE phase, rather than in the PE phase. These results indicated that miRNAs influence the establishment of endometrial receptivity, providing experimental basis for the detection of endometrial receptivity in dairy goats. In addition, miR‐100‐5p was significantly downregulated in the RE phase in the differential expression profile of miRNAs, demonstrating that miR‐100‐5p was involved in regulating the establishment of endometrial receptivity during embryo implantation in dairy goats. The results of qRT‐PCR were identical with the sequencing results that the expression of miR‐100‐5p was lower in the RE comparing with that in the PE, but the function of miR‐100‐5p in the endometria of dairy goats is rare.

CircRNAs, crucial regulators, have attracted much attention in recent years because of its specificity of expression and complexity of regulation, as well as its important role in disease occurrence.^15^ CircRNAs regulate miRNAs by competitively binding with other endogenous RNAs, so they play a critical role in epigenetic, transcriptional and post‐transcriptional regulation.^16^ For instance, circLMO7 regulated myoblasts differentiation and survival by acting as a sponge for miR‐378a‐3p.^17^ Circ‐8073 regulated CEP55 by acting as a ceRNA sponge for miR‐449a.^18^ However, the regulation effect of specific CircRNAs on the function of miR‐100‐5p in endometrial stromal cells (ESCs) remains unknown. In the present study, we demonstrated that miR‐100‐5p‐induced apoptosis of goat ESCs through targeting Circ‐9110/miR‐100‐5p/HOXA1 axis, resulting in performing its embryo implantation function.

## 2 MATERIALS AND METHODS

### 2.1 Animals and endometrial sample collection

The dairy goats were collected according to the experiment guide with the No. 5 Proclamation of the Ministry of Agriculture, P. R. China. The sample collection process followed the rule of the approval of the Review Committee for the Use of Animal Subjects of Northwest A&F University. The experimental samples were collected in accordance with the approval of the Review Committee for the Use of Animal Subjects of Northwest A&F University referring to the animals and care protocol. The specific operations were as follows: the endometrias of the experimental goats were taken from the inner walls of their uterine cavities in the pre‐receptive phase (*n* = 10) and receptive phase (*n* = 10).^13^, ^14^ After all the samples of different groups were washed with phosphate‐buffered saline (PBS) and marked, they were immediately brought back to the laboratory.

### 2.2 Cell culture, purification and treatment

Primary ESCs were isolated with trypsin digestive method, then differential centrifugation and difference tempo adherence were employed. Cells were cultured in 6‐well plates containing DMEM/F12 medium with 10% FBS and 1% penicillin‐streptomycin for 24 h, then the cellular morphology was observed under a light microscope. Cell purification and identification were evaluated immunocytochemically according to a previous report.^19^

### 2.3 Vector construction and transfection

The full length of Circ‐9110 was extended by PCR and then cloned in a pcD2.1‐cir vector (Geneseed Biotech, Guangzhou, China). Given that the CDSs of HOXA1 X1 were not amplified in dairy goats, the CDSs of HOXA1 X2 were cloned and inserted in pcDNA3.1(+) vectors (Promega, Madison, USA).

MiR‐100‐5p mimic, NC mimic, miR‐100‐5p inhibitor, NC inhibitor, Circ‐9110‐siRNA and HOXA1‐siRNA were synthesized with Ribobio (Guangzhou, China). The ESCs were seeded at a density of 7.5 × 10^5^ cells/well in 6‐well plates and then transfected at 60% confluency with miR‐100‐5p mimic, miR‐100‐5p inhibitor, NC mimic or NC inhibitor, pcDNA3.1, pcDNA3.1‐HOXA1 and pcD2.1‐ciR or pcD2.1‐circ‐9110 at 100 nM final concentrations by using Lipofectamine 2000 (Invitrogen, USA) according to the manufacturer's specifications.

### 2.4 Quantitative real‐time PCR and Western blot

The total RNAs of the tissues and ESCs were extracted using Trizol reagent (TaKaRa, Dalian, China) and converted to cDNA by using a Prime Script RT reagent kit with gDNA eraser (Genstar, Beijing, China) according to the manufacturer's specifications. qRT‐PCR was performed with SYBR Green PCR Master Mix (TaKaRa, Dalian, China). The PCR procedures were as follows: initial denaturation for 30 s at 95°C, denaturation for 5 s at 95°C, followed by 40 cycles of annealing for 30 s at 60°C and extending for 50 s at 72°C. At the end of the total runs, a melting‐curve analysis (95°C for 15 s and 60°C for 1 min at 0.5°C/5 s until 95°C) was performed for ensuring the specificity of amplification. All the PCR primers used in this study are listed in Table 1. Intracellular expression of objective gene and β‐actin (as control) was quantitated by qRT‐PCR. Data were analysed by using 2^−△△CT^ values.

**TABLE 1:** qRT‐PCR Primers used in the present study

Gene	GenBank accession no	Primer sequences (5′→3′)
Circ‐9110(Check2)		F:CG *CTCGAG* CCCGGGAGTCGCTAGAGTTG R:AT *GCGGCCGC* CTCCAACCACAGTGGAGGCA
Circ‐9110(pc2.1)		F:GG *GGTACC* TGAAATATGCTATCTTACAGAGCCACCAACCAAATACCAAATCTCC R:CG *GGATCC* TCAAGAAAAAATATATTCACCTTCAGAACCTTGAGATAGGGCAGCC
Circ‐9110(qPCR)		F: CCCAGCCCCATATCCAGTGG R: CAACTCTAGCGACTCCCGGG
miR‐100‐5p‐RT Primer	–	GTCGTATCCAGTGCAGGGTCCGAGGTATTCGCACTGGATACGACCACAAG
miR‐100‐5p‐FW	–	CCGCGAACCCGTAGATCCGAA
REVERSE Primer	–	ATCCAGTGCAGGGTCCGAGG
U6	–	F:CTCGCTTCGGCAGCACA
		R:AACGCTTCACGAATTTGCGT
HOXA1(Check2)	–	F:AG *CTCGAG* ACCAGCTTCTTCCTTCCACAGT
		R:AT *GCGGCCGC* ATATATTTATTACAGACATCTTAAGACCCGT
HOXA1(qPCR)	–	F:CCAGCCGACAACCTATCAGACTTC
		R:AGGCTTCTTGGTGGTTCTGCTTC
HOXA1(pcDNA3.1)	–	F:GCG *AAGCTT* ATGGACAATGCAAGAATGAGCTCCTTC
		R:CG *GGATCC* TCAAAGGTCTGTGCTGGAGAAGAGG
β‐Actin	XM_018039831.1	F:GATCTGGCACCACACCTTCT
		R:GGGTCATCTTCTCACGGTTG

Note

The characters with underscore were restriction enzyme cutting site of Xho I and Not I for constructing psiCHECK2. The italicized characters with underscore were restriction enzyme cutting site of BamH I and Hind Ⅲ for constructing pcDNA3.1. The italicized characters with underscore were restriction enzyme cutting site of Kpn I and BamH I for constructing pCD2.1‐ciR.

Whole cell lysates were prepared in M‐PER mammalian protein extraction reagent (Thermo Fisher) and complete protease inhibitor cocktail (1:100). Equal amount (30–50 μg/well) of protein samples was separated on SDS‐PAGE and transferred to PVDF membranes. The membranes were first blocked with 10% non‐fat dry milk and incubated with the primary antibodies. The antibodies applied were shown in Table 2. After incubation with appropriate secondary antibodies, the immunoblots were incubated with ECL plus Western blotting substrate (Thermo Fisher). The results were imaged using a gel image analysis system (Bio‐Rad, USA) according to the manufacturer's instructions.

**TABLE 2:** Antibodies used in the present study

Name	Manufacturer	Product number
Cytokeratin 20	Abcom, America	Ab76126
Vimentin	Boster Co, Wuhan, China	BM0135
BAX	Beyotime, Shanghai, China	AB026
BCL2	Beyotime, Shanghai, China	AB112
Caspase3	Cell Signaling, America	#9662
p‐PI3K p110 beta (Ser1070)	Bioss, Beijing, China	bs‐6417R
PI3K p110 beta	Bioss, Beijing, China	bs‐6423R
AKT	Cell Signaling, America	#9272
p‐AKT (Ser473)	Cell Signaling, America	#9271
p‐mTOR (S2448)	Boster, Wnhan, China	BM4840
mTOR	Boster, Wnhan, China	BM4182
HOXA1	BBI, Shanghai, China	D152305
β‐Actin	Beyotime, Shanghai, China	AA128
ERK1/2	ABclonal, Wuhan, China	A16686
p‐ERK1/2	ABclonal, Wuhan, China	AP0472
RAS	Bioss, Beijing, China	bs‐1515R
HRP‐labelled Goat Anti‐Rabbit IgG (H + L)	Beyotime, Shanghai, China	A0208
HRP‐labelled Goat Anti‐Mouse IgG (H + L)	Beyotime, Shanghai, China	A0216

### 2.5 Luciferase assay

For the production of reporter structures for the luciferase assay, the 3′‐UTRs of HOXA1 and Circ‐9110 that was bound with miR‐100‐5p were cloned and inserted into the psiCHECKTM‐2 vectors (Promega, Madison, USA). The mutated plasmids with mutated target sites (psiCHECK2‐Mut) were constructed. The constructs produced by the primers were verified by sequencing analysis. The primer sequences were shown in Table 1. NC, miR‐100‐5p mimic, NC inhibitor and miR‐100‐5p inhibitor were individually co‐transfected with a wild‐type plasmid (psiCHECK2‐WT) into 293T cells for 48h, and the same procedure was conducted with mutated plasmid (psiCHECK2‐Mut). Luciferase assay was performed and analysed as previously described ^19^.

### 2.6 Cell proliferation, cycle and apoptosis assay

ESCs were seeded at ~2000 cells/well in 96‐well plates. After 24 h, the cells were incubated with or miR‐100‐5p mimic alone, miR‐100‐5p inhibitor, NC mimic or NC inhibitor, pcDNA3.1, pcDNA3.1‐HOXA1 and pcD2.1‐ciR or pcD2.1‐circ‐9110 for 24 and 48 h. Cell viability was determined using CCK‐8 assay (EnoGene, Nanjing, China), and cell proliferation was further investigated using EdU (Ribobio, Guangzhou, China) according to the manufacturer's specifications.^20^ After cells (2 × 10^5^ cells/well) with different treatment in 6‐well plate for 24 h, cells were detached using 0.05% trypsin from the plates and washed. The cell apoptosis detection and cell cycle analysis were performed using Annexin V‐FITC/PI apoptosis kit (TransGen Biotech, Beijing, China) and cell cycle staining kit (Liankebio, Hangzhou, China) with flow cytometer, respectively. Cell apoptosis was analysed using the FlowJo software.

### 2.7 Animal experiments

To further investigate the expression pattern of miR‐100‐5p in early pregnancy in vivo, the ICR mice models were established. Three ICR males (8–10 weeks old) and six ICR females (6–8 weeks old) were caged in the evening (6:00 p.m.) at a ratio of 1:2 to induce mating, and the morning of vaginal plug visualization was designated as day 1 of pregnancy (D1). At day 3 of pregnancy (D3), three mice were anaesthetized at 08:00 h and 10 μl solution containing 10 nmol miR‐100‐5p agomir (RiboBio, Guangzhou, China) was injected into the left horn of uterus of each mouse, while the right horn was injected with agomir NC. The experimental mice were separated into two groups, four mice uterus specimens were collected at day 4.5 of pregnancy (D4.5) and then fixed with 2.5% glutaraldehyde. Other four mice were dissected at day 9 of pregnancy (D9) of pregnancy to observe the number of embryo implantation.

### 2.8 Scanning electron microscopy

Uterine tissues were fixed in 2.5% glutaraldehyde in phosphoric acid buffer solution lasting more than 2 h. The samples were rinsed three times with 0.1 M phosphoric liquid for 2 h, and each for 15 min. After treatment with 1% osmium tetroxide for 2 h, the cells were rinsed three times with 0.1 M phosphoric acid solution, each for 15 min. They were dehydrated with a series of incubations in ethanol. Dehydration was continued by incubations in 95% ethanol, followed by absolute ethanol. The change of uterine tissues preincubated with miR‐100‐5p agomir was detected and photographed by Scanning electron microscope (SEM) (SU8100, Hitachi, Japan).

### 2.9 Immunohistochemistry

Slides cut from uterine tissues underwent drying, rehydration, antigen retrieval and permeation before the samples were blocked in goat serum for 20 min and incubated in the primary antibody (*HOXA1*, 1:1000) then in the second antibody. Colour development was performed with a DAB Substrate kit (Solarbio, DA1010, Beijing, China) and counterstained with haematoxylin (Solarbio, H8070, Beijing, China).

### 2.10 Statistical analysis

Data were analysed using SPSS 17.0 (SPSS Inc., Chicago, IL, USA). t‐Test or one‐way ANOVA, followed by the least significant difference, was used to compare the differences between two or among three groups, respectively. Each result was expressed as the means ± standard error. Differences were considered significant and very significant at *p* < 0.05 and *p* < 0.01, respectively.

## 3 RESULTS

### 3.1 Endometrial cell isolation and identification

The ESCs were isolated from the uterus by the previous description.^19^ After 48 h of culture, the ESCs reached sub‐confluence and exhibited a fibroblast‐like appearance (Figure S1A). As shown in Figure S1B‐D, we carried out morphological analysis of the cells in the RE and PE with DAPI staining, clearly presenting that the nuclei of cells displayed blue. The cell membrane which turned green after staining with vimentin was negative after staining with cytokeratin (Figure S1E–H).

### 3.2 MiR‐100‐5p inhibited the proliferation of ESCs and induced ESCs apoptosis in vitro

**FIGURE 1:**
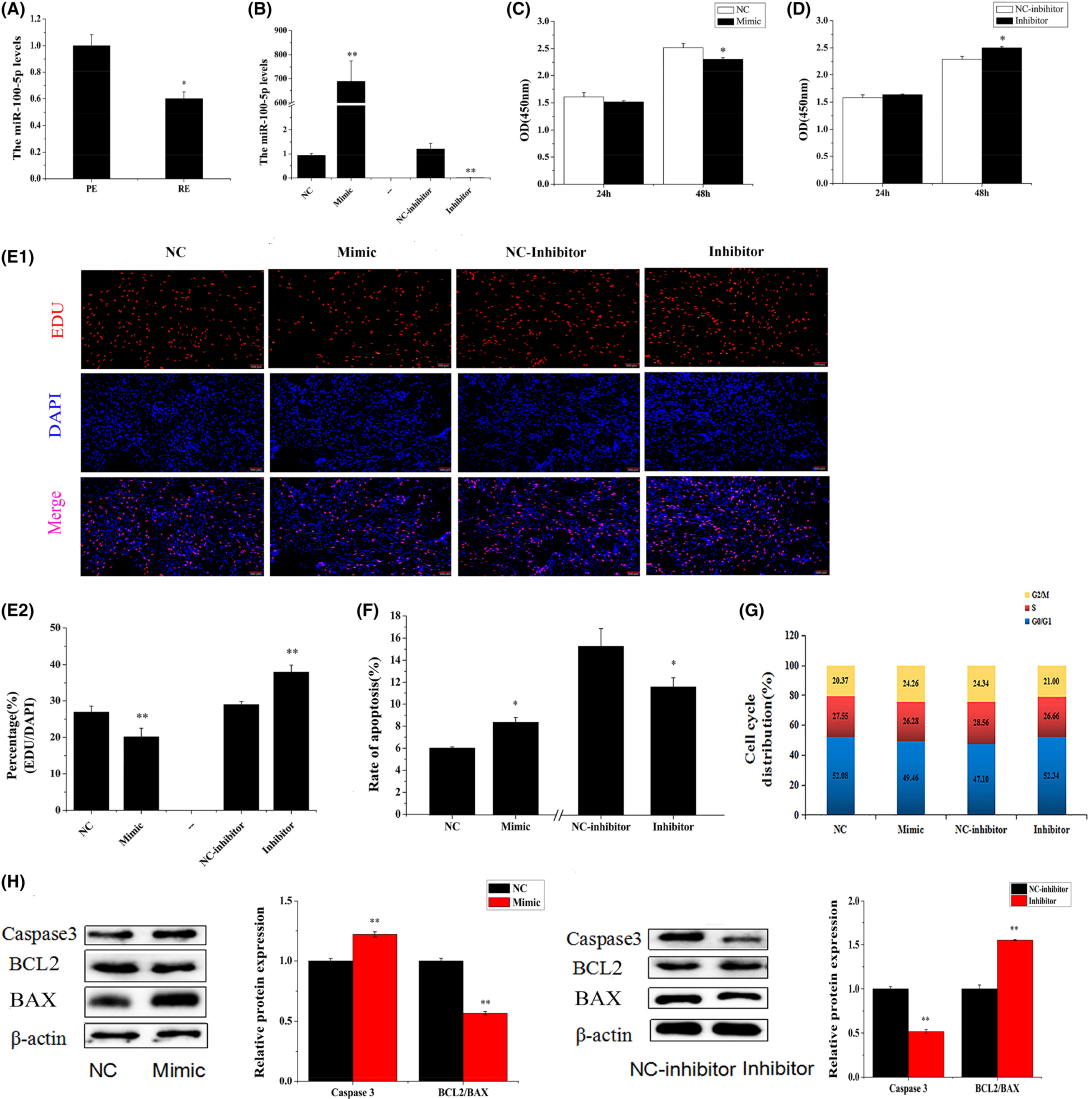
MiR‐100‐5p inhibited ESCs proliferation and induced ESCs apoptosis *in vitro*. Note: (A) The miR‐100‐5p expression levels in endometrium PE and RE. (B) Efficiency of miR‐100‐5p transfection in the ESCs. (C) ESCs viability after miR‐100‐5p transfection (D) ESCs were transfected with NC inhibitor or miR‐100‐5p inhibitor, and cell proliferation was assessed using the cell counting kit‐8(CCK‐8) assay. (E) Cell proliferation indices were assessed after treatment with EdU. Scale bar = 100 μm. (F) ESCs were transfected with NC, miR‐100‐5p, NC inhibitor or miR‐100‐5p inhibitor, and the apoptosis analysis of ESCs was performed with FCM. (G) ESCs were transfected with NC, miR‐100‐5p, NC inhibitor or miR‐100‐5p inhibitor, and cell phases were analysed by FCM. (H) Caspase3, BCL2 and BAX protein levels in the ESCs that were transfected with NC, miR‐100‐5p, NC inhibitor or miR‐100‐5p inhibitor. Protein levels were normalized to β‐actin from the same lane. The values are shown as mean ± SEM for three individuals. ** indicates that *p *< 0.01; * indicates that *p *< 0.05

**FIGURE 2:**
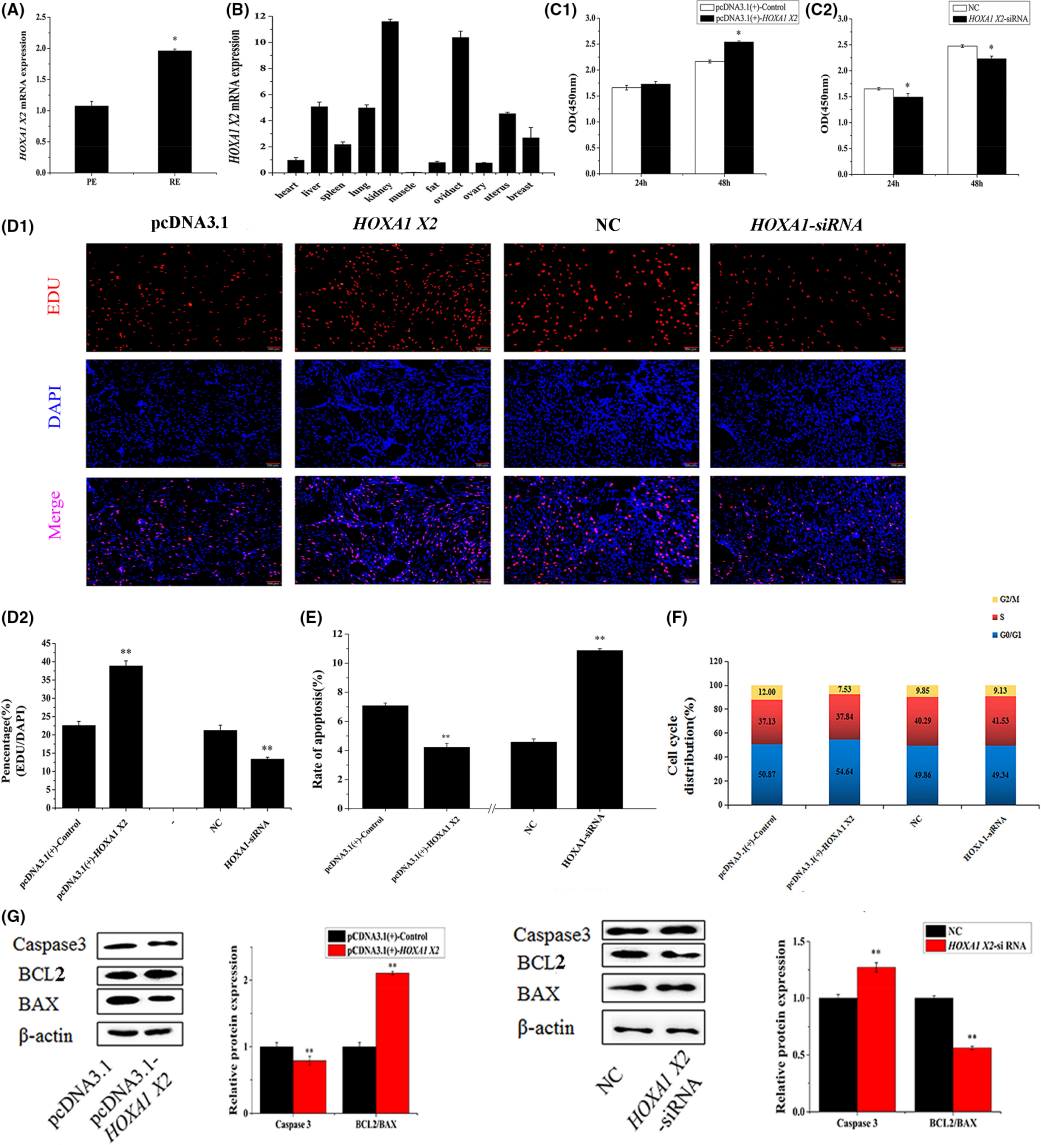
*HOXA1* promoted ESCs proliferation inhibited ESCs apoptosis in vitro. Note: (A) *HOXA1* levels in endometrium PE and RE. (B) *HOXA1* was expressed in various dairy goat tissues. (C) ESCs were transfected with pcDNA3.1, pcDNA3.1(+)‐*HOXA1*, NC or *HOXA1*‐siRNA, and cell viability was assessed using the cell counting kit‐8(CCK‐8) assay. (D) Cell proliferation indices were assessed after treatment with EdU. Scale bar = 100 μm. (E) ESCs were transfected with pcDNA3.1, pcDNA3.1(+)‐*HOXA1*, NC or *HOXA1*‐siRNA, and the apoptosis analysis of ESCs was performed with FCM. (F) ESCs were transfected with pcDNA3.1, pcDNA3.1(+)‐HOXA1, NC, or *HOXA1*‐siRNA, and cell phases were analysed by FCM. (G) The Caspase3, BCL2 and BAX protein levels in the ESCs that were transfected with pcDNA3.1, pcDNA3.1(+)‐*HOXA1*, NC or *HOXA1*‐siRNA. The protein levels were normalized to β‐actin from the same lane. The values are shown as mean ± SEM for three individuals. ** indicates that *p* < 0.01; * indicates that *p *< 0.05

Stem‐loop qRT‐PCR was used to detect the efficiency of miR‐100‐5p transfection in the ESCs. These results indicated that miR‐100‐5p expression was prominently improved by the miR‐100‐5p mimic but significantly inhibited by the miR‐100‐5p inhibitor (*p *< 0.01, Figure 1B). The function of miR‐100‐5p on ESCs was evaluated after transfecting the miR‐100‐5p mimic, NC, miR‐100‐5p inhibitor and NC inhibitor into ESCs. The CCK‐8 analysis showed that the miR‐100‐5p treatment with 48 h had significantly suppressed ESCs viability (*p *< 0.05, Figure 1C) but it had no obvious effect with 24 h. The ESCs viability was slightly increased with the increasing of miR‐100‐5p inhibitor treatment duration (from 24 to 48 h) (*p *< 0.05, Figure 1D). The EdU assay further revealed that miR‐100‐5p reduced ESC proliferation, whereas the miR‐100‐5p inhibitor raised ESCs proliferation (Figure 1E). After being treated with miR‐100‐5p and miR‐100‐5p inhibitor, ESCs viability was measured by flow cytometry (FCM). Apoptosis rate occurred in 8.18% cells after 48 h of MiR‐100‐5p treatment induced, which was higher than that of the control (5.86%). After the ESCs were treated with the miR‐100‐5p inhibitor, the rate of apoptotic cells decreased from 17.29% to 11.38% (Figure S2A). The results showed that miR‐100‐5p significantly induced ESCs apoptosis and the miR‐100‐5p inhibitor obviously depressed the apoptosis proportion of ESCs (*p *< 0.01, Figure 1F). The effect of miR‐100‐5p on the cell cycle of ESCs was assessed by TCM. The results indicated that the NC treatment yielded 20.37% cells at the G2/M phase, whereas the miR‐100‐5p treatment caused the accumulation of the ESCs at the G2/M phase with 24.26% (Figures 1G and 2B). The miR‐100‐5p inhibitor treatment decreased the percentage of the G2/M cells (21%) compared with the percentage obtained through the NC inhibitor treatment (24.34%) (Figures 1G and 2B). The protein expression levels of BAX, BCL2 and Caspase3 were also detected by Western blot. The results suggested that miR‐100‐5p significantly improved Caspase3 expression and significantly reduced the BCL2/BAX value in the ESCs in vitro (*p *< 0.01, Figure 1H). Conversely, the miR‐100‐5p inhibitor significantly raised the BCL2/BAX value (*p *< 0.01, Figure 1H). The results showed that miR‐100‐5p may induce ESCs apoptosis by reducing the BCL2/BAX value. The all above data illustrated that miR‐100‐5p could inhibit the proliferation of ESCs, induce ESCs apoptosis and has a function opposite that of the miR‐100‐5p inhibitor.

### 3.3 HOXA1 is a target gene of miR‐100‐5p

To find the target genes of miR‐100‐5p, we predicted the potential target genes using two publicly available programs (Targetscan 7.0 and miRanda). The candidates of target genes were validated using a psiCHECK2 reporter (WT) and mutated plasmid (MUT) (Figure S3A). The results showed that the miR‐100‐5p mimic significantly reduced luciferase activity in WT‐*HOXA1*‐3’UTR compared with the activity in NC (*p *< 0.05), whereas no significant activity difference was found in the MUT‐HOXA1‐3’UTR (Figure S3B). Furthermore, the *HOXA1* expression was significantly suppressed after the miR‐100‐5p treatment (*p *< 0.05) but significantly increased after the miR‐100‐5p inhibitor treatment (*p *< 0.01, Figure S3C). The relative protein expression of *HOXA1* was considerably decreased in the miR‐100‐5p mimic‐transfected ESCs (*p *< 0.01), and miR‐100‐5p inhibitor slightly increased the amount of ESCs in vitro (Figure S3D). In summary, these results suggested that miR‐100‐5p targeted *HOXA1* and downregulated *HOXA1* expression in ESCs in vitro.

### 3.4 HOXA1 promoted the cell proliferation and inhibited the apoptosis of ESCs in vitro

The level of *HOXA1* expression significantly was much higher in the RE compared with those in the PE (*p *< 0.05, Figure 2A). *HOXA1*, a target gene of miR‐100‐5p, was widely expressed in various dairy goats’ tissues. The highest expression level was found in the kidneys, followed (in order) by the expression levels in the oviducts, livers, lungs, uterus, breasts, spleens, hearts, ovaries, fats and muscles (Figure 2B). The efficiency of *HOXA1* transfection in the ESCs was determined through qRT‐PCR analysis. The results indicated that the transfection *HOXA1* markedly raised the mRNA and protein expression of *HOXA1* in the ESCs (*p *< 0.01). *HOXA1* knocked down by siRNA saliently reduced *HOXA1* mRNA and protein expression (*p *< 0.05, Figure S4A–C).

The function of *HOXA1* on the ESCs was investigated, pcDNA3.1(+)‐Control, pcDNA3.1(+)‐*HOXA1*, NC and *HOXA1*‐siRNA were separately transfected into the ESCs. CCK‐8 analysis indicated that *HOXA1* treatment for 48 h caused significant increase in ESCs viability (*p *< 0.05), but the effect of 24 h *HOXA1* treatment was not significant. By contrast, both 24 and 48 h *HOXA1*‐siRNA treatment obviously decreased ESCs viability (*p *< 0.05, Figure 2C). Besides, EdU assays revealed that *HOXA1* and *HOXA1*‐siRNA significantly promoted and reduced ESCs proliferation in vitro, respectively (*p *< 0.01, Figure 2D).

Annexin V‐binding assay was used for the quantitative analysis of the effects of *HOXA1* on ESC apoptosis induction. As presented in Figure S3A, *HOXA1* reduced ESC apoptosis rate to 4.87% compared with the pcDNA3.1(+)‐Control (7.89%), whereas the *HOXA1*‐siRNA increased the apoptosis rate to 12.57% in the ESCs compared with NC (4.35%). The results showed that the apoptosis proportion of the ESCs was considerably decreased after the introduction of *HOXA1*, and the apoptosis rate of ESCs was markedly increased after the introduction of *HOXA1*‐siRNA (*p* < 0.01, Figure 2E and Figure S5A). We further analysed the cell cycle distribution of the cell population. The results in Figure 2F and Figure S5B showed that the pCDNA3.1(+)‐*HOXA1* yielded 7.53% cells at the G2/M phase of the cell cycle, but the pCDNA3.1(+)‐Control treatment resulted in the accumulation of the ESCs at the G2/M phase (12.00%). No salient difference in cell cycle distribution was found between the NC and *HOXA1*‐siRNA treatments. Some apoptosis‐related genes were detected by Western blot when *HOXA1* was overexpressed or knocked down in the ESCs in vitro. The protein expression levels of BCL2/BAX and Caspase 3 were considerably increased and decreased, respectively, after the introduction of *HOXA1* (*p* < 0.01). The protein expression levels of BCL2/BAX and Caspase 3 were substantially decreased and increased, respectively, after the introduction of *HOXA1*‐siRNA (*p* < 0.01, Figure 2G).

### 3.5 Circ‐9110 served as a sponge for miR‐100‐5p

**FIGURE 3:**
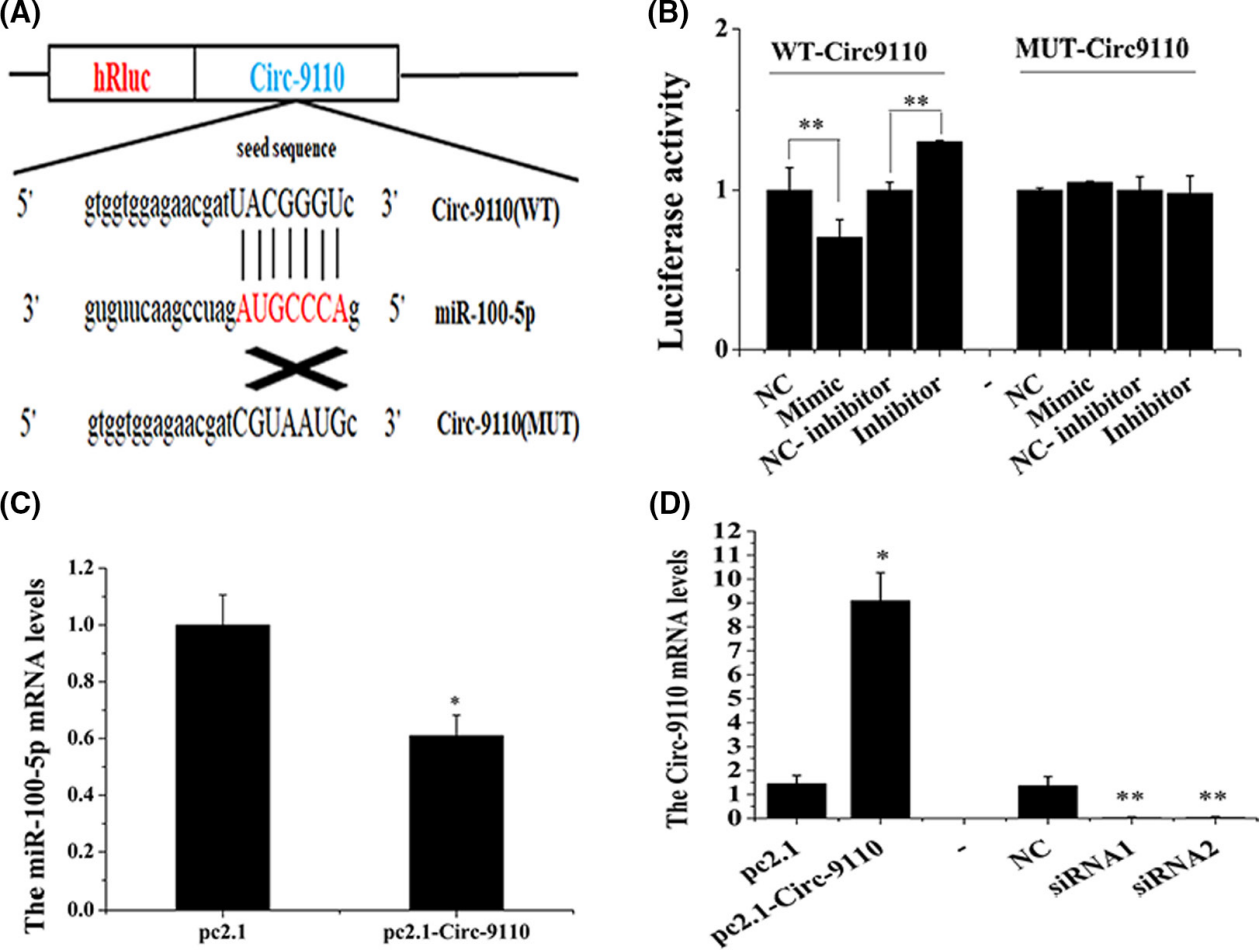
Circ‐9110 acted as a miRNA sponge and thereby decreased miR‐100‐5p levels in the ESCs Note: (A) Schematic diagram illustrating the design of luciferase reporters with the WTCirc‐9110 or site‐directed mutant (MUT‐Circ‐9110). The nucleotides in red represent the ‘seed sequence’ of miR‐100‐5p. (B) The luciferase reporter assay of 293T cells co‐transfected with WTCirc‐9110 or MUT‐Circ‐9110 and miR‐100‐5p mimic, NC, miR‐100‐5p inhibitor or NC inhibitor. (C) Circ‐9110 decreased miR‐100‐5p mRNA level in the ESCs. (D) The levels of Circ‐9110 after transfection with Circ‐9110 (pc2.1‐Circ‐9110) or Circ‐9110 siRNAs. The values are shown as mean ± SEM for three individuals. ** indicates that *p *< 0.01; * indicates that *p *< 0.05

The miR‐100‐5p was predicted to functionally target Circ‐9110 by bioinformation analysis. The predicted binding sites between Circ‐9110 and miR‐100‐5p were validated using a constructed WT and the mutated plasmid (MUT) (Figure 3A). The luciferase activity of the WTCirc‐9110 + miR‐100‐5p mimic group was significantly lower than that of the WTCirc‐9110 + NC group (*p *< 0.01). The luciferase activity of the WTCirc‐9110 + miR‐100‐5p inhibitor group was significantly higher than that of the WTCirc‐9110 + NC inhibitor group (*p *< 0.01), and the luciferase activity of the MUT‐Circ‐9110 + miR‐100‐5p mimic group had no changed (Figure 3B). Furthermore, the qRT‐PCR results showed that Circ‐9110 could inhibit miR‐100‐5p expression in ESCs (Figure 3C, *p* < 0.05). siRNAs were used to silence the expression of Circ‐9110. qRT‐PCR was used in detecting the efficiency of Circ‐9110 and two siRNA transfection in the ESCs. The results indicated that overexpressed Circ‐9110 markedly improved the mRNA expression of Circ‐9110 in the ESCs (*p *< 0.05) and siRNA1 and siRNA2 significantly reduced Circ‐9110 mRNA expression (Figure 3D, *p* < 0.01).

### 3.6 Circ‐9110 promoted the cell proliferation and inhibited the apoptosis of ESCs in vitro

**FIGURE 4:**
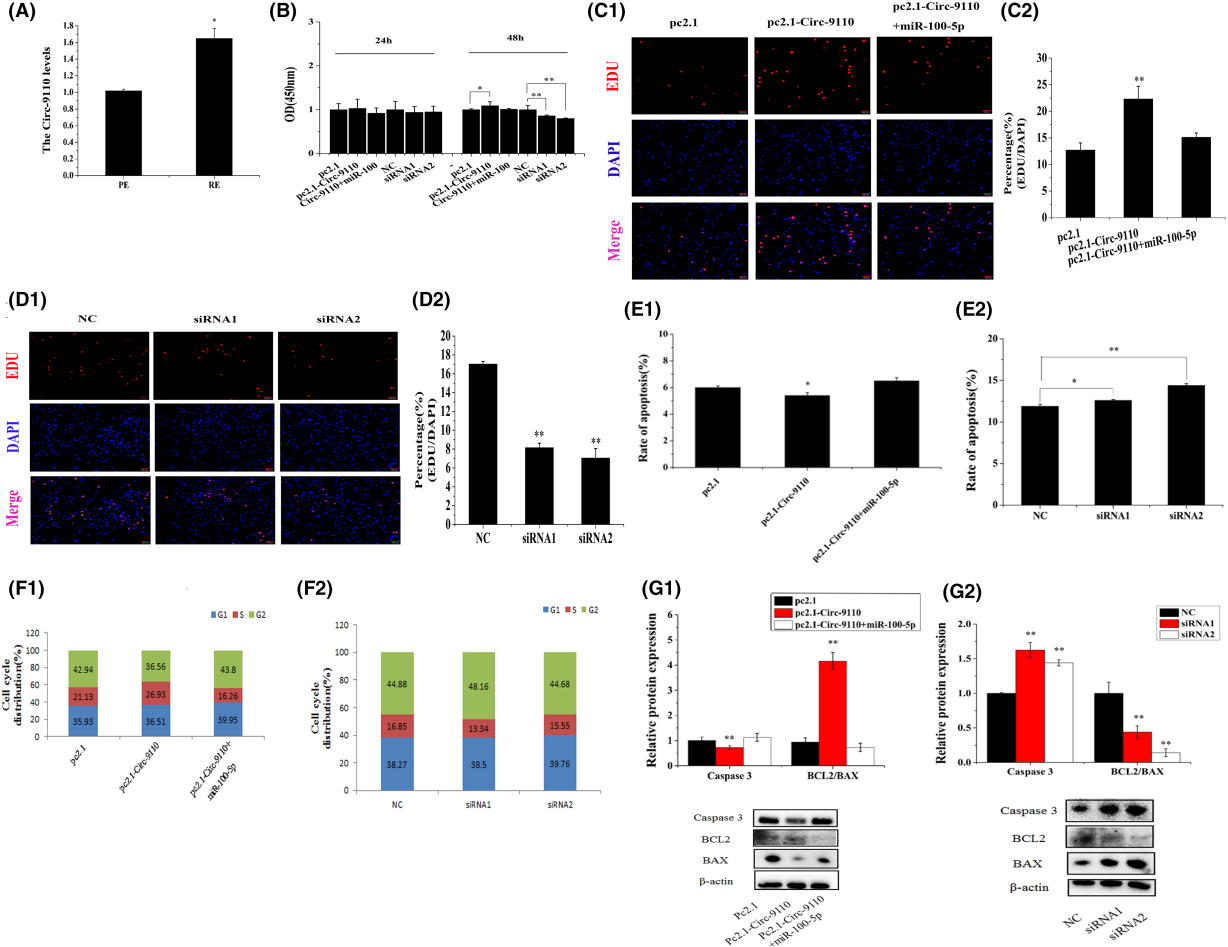
Circ‐9110 promoted ESCs proliferation inhibited ESCs apoptosis in vitro. Note: (A) Circ‐9110 levels in endometrium PE and RE. (B) ESCs were transfected with pc2.1, pc2.1‐Circ‐9110, pc2.1‐Circ‐9110+miR‐100‐5p, NC, siRNA1 or siRNA2, and cell viability was assessed using the cell counting kit‐8(CCK‐8) assay. (C, D) Cell proliferation indices were assessed after treatment with EdU. (E1) ESCs were transfected with pc2.1, pc2.1‐Circ‐9110 or pc2.1‐Circ‐9110+miR‐100‐5p, and the apoptosis analysis of ESCs was performed with FCM. (E2) ESCs were transfected with NC, siRNA1 or siRNA2, and the apoptosis analysis of ESCs was performed with FCM. (F1) ESCs were transfected with pc2.1, pc2.1‐Circ‐9110 or pc2.1‐Circ‐9110+miR‐100‐5p, and cell phases were analysed by FCM. (F2) ESCs were transfected with NC, siRNA1 or siRNA2, and the cell phases were analysed by FCM. (G1) Caspase3, BCL2 and BAX protein levels in ESCs transfected with pc2.1, pc2.1‐Circ‐9110 or pc2.1‐Circ‐9110+miR‐100‐5p. (G2) Caspase3, BCL2 and BAX protein levels in ESCs that were transfected with NC, siRNA1 or siRNA2. Protein levels were normalized to β‐actin. Scale bar = 100 μm. The values are shown as mean ± SEM for three individuals. ** indicates that *p *< 0.01; * indicates that *p *< 0.05

Circ‐9110 expression levels were much higher in RE compared with that in PE (*p *< 0.05, Figure 4A). For the investigation of the function of Circ‐9110 on the ESCs, pc2.1 pc2.1‐Circ‐9110, pc2.1‐Circ‐9110+miR‐100‐5p, NC, siRNA1 and siRNA2 were transfected into the ESCs. CCK‐8 analysis results suggested that ESCs viability had no obvious change after the 24 h pc2.1‐Circ‐9110 treatment but significantly increased after the 48 h pc2.1‐Circ‐9110 treatment (*p *< 0.05). ESCs viability was significantly suppressed after the 48 h siRNA1 and siRNA2 treatments (*p *< 0.01) but showed non‐significant effect after 24 h siRNA1 and siRNA2 treatments (Figure 4B). EdU assays manifested that Circ‐9110 could promote ESCs proliferation (Figure 4C, *p* < 0.01) and miR‐100‐5p could weaken the effect of Circ‐9110 on ESCs proliferation. By contrast, Circ‐9110 siRNA1 and siRNA2 could reduce ESCs proliferation (Figure 4D, *p* < 0.01).

Annexin V‐binding assay was used for the quantitative analysis of the effects of Circ‐9110 on ESCs apoptosis induction. As presented in Figure S6A and Figure 4E1, Circ‐9110 reduced ESCs apoptosis rate (*p* < 0.05). Circ‐9110 siRNA1 and siRNA2 promoted ESC apoptosis rate (Figure S6B and Figure 4E2, *p* < 0.05). We further analysed the cell cycle distribution of the cell population. The results in Figure S6C,D and Figure 4F showed that Circ‐9110 increased the number of the ESCs at the S phase but decreased the proportion of the ESCs at the G2 phase. Otherwise, siRNA1 increased the number of ESCs at the G2 phase but decreased the proportion of ESCs at the S phase. However, no salient difference in cell cycle distribution was found between NC and the siRNA2 treatments.

Some apoptosis‐related genes were detected by Western blot in the overexpressed or knocked down Circ‐9110 in ESCs in vitro. Marked increase and decrease in the relative protein expression levels of BCL2/BAX and Caspase 3, respectively, were observed after the introduction of Circ‐9110 (Figure 4G1, *p*<0.01). Meanwhile, significant decreasement and increasement in the relative protein expression levels of BCL2/BAX and Caspase 3, respectively, were found after the treatment of siRNA1 and siRNA2 (Figure 4G2, *p *< 0.01).

### 3.7 MiR‐100‐5p and HOXA1 regulated the PI3K/AKT/mTOR and ERK1/2 signal pathways in ESCs

**FIGURE 5:**
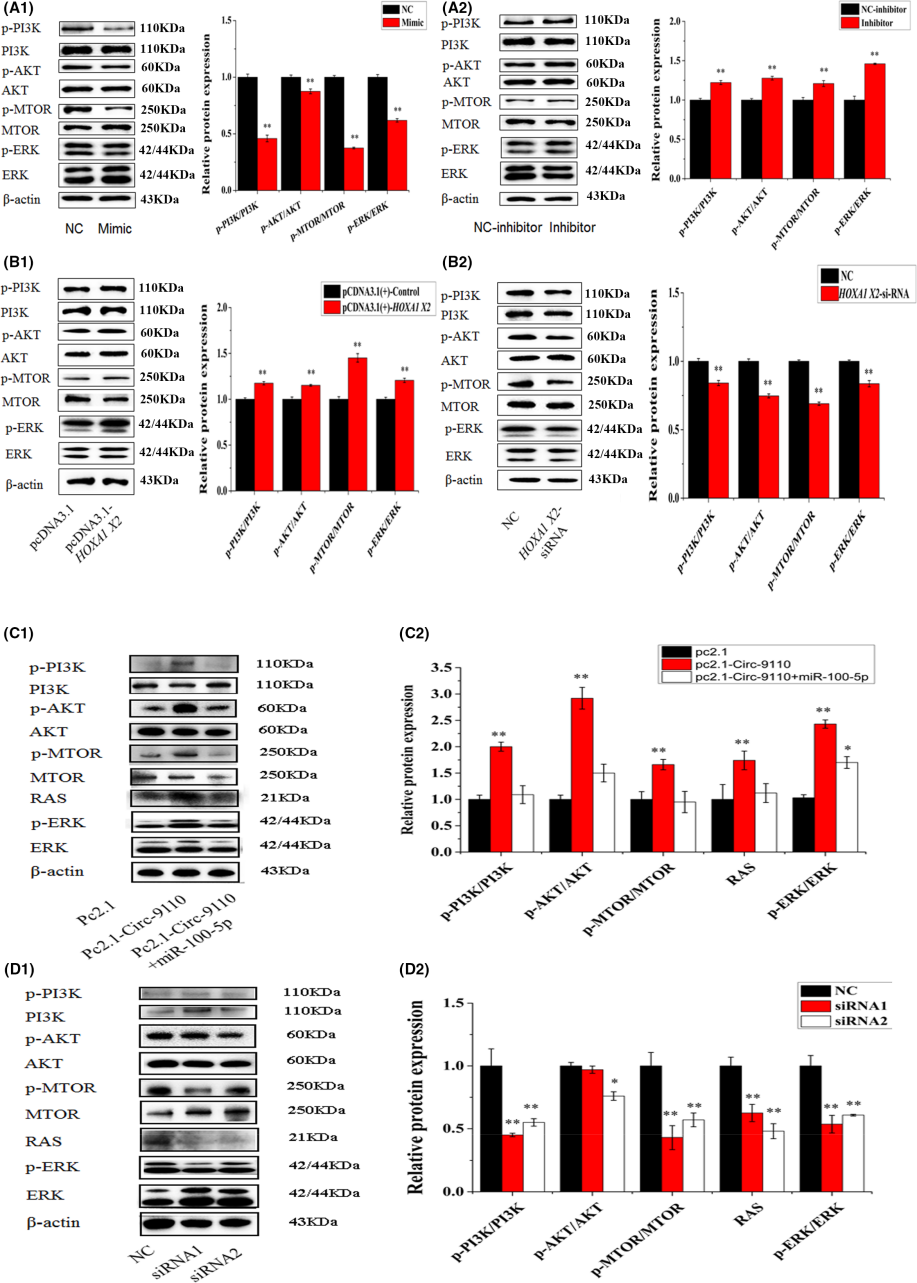
miR‐100‐5p, HOXA1 and Circ‐9110 regulated the PI3K/AKT/mTOR and ERK1/2 signal pathways in the ESCs. Note: (A) p‐PI3K, PI3K, p‐AKT, AKT, p‐MTOR, MTOR, p‐ERK and ERK protein levels in the ESCs transfected with miR‐100‐5p mimic or miR‐100‐5p inhibitor. (B) p‐PI3K, PI3K, p‐AKT, AKT, p‐MTOR, MTOR, p‐ERK and ERK protein levels in the ESCs transfected with pcDNA3.1(+)‐HOXA1 or HOXA1‐siRNA. (C) p‐PI3K, PI3K, p‐AKT, AKT, p‐MTOR, MTOR, RAS, p‐ERK and ERK protein levels in the ESCs transfected with pc2.1, pc2.1‐Circ‐9110 or pc2.1‐Circ‐9110+miR‐100‐5p. (D) p‐PI3K, PI3K, p‐AKT, AKT, p‐MTOR, MTOR, RAS, p‐ERK and ERK protein levels in the ESCs transfected with NC, siRNA1 or siRNA2. Protein levels were normalized to β‐actin. The values are shown as mean ± SEM for three individuals. ** indicates that *p *< 0.01

The relative proteins involved in the PI3K/AKT/mTOR and ERK signal pathways were detected and used in estimating the effects of miR‐100‐5p and *HOXA1* on ESCs survival. As shown in Figure 5A,B, in ESCs, the relative protein expression levels of p‐PI3K/PI3K, p‐AKT/AKT, p‐mTOR/mTOR and p‐ERK1/2/ERK1/2 decreased substantially (*p* < 0.01) after the miR‐100‐5p treatment, but that of p‐PI3K/PI3K, p‐AKT/AKT, p‐mTOR/mTOR and p‐ERK1/2/ERK1/2 increased considerably (*p* < 0.01) after the miR‐100‐5p inhibitor treatment. The relative protein expression levels of p‐PI3K/PI3K, p‐AKT/AKT, p‐mTOR/mTOR and p‐ERK1/2/ERK1/2 showed obvious increase (*p* < 0.01) after the introduction of HOXA1. The relative protein expression levels of p‐PI3K/PI3K, p‐AKT/AKT, p‐mTOR/mTOR and p‐ERK1/2/ERK1/2 significantly decreased (*p* < 0.01) after HOXA1 was knocked down. The above results suggested that miR‐100‐5p, which targets HOXA1, can regulate cell proliferation and apoptosis by the PI3K/AKT/mTOR and ERK1/2 signal pathways in ESCs in vitro.

### 3.8 Circ‐9110 regulated the PI3K/AKT/mTOR and ERK1/2 signal pathways in ESCs

The relative protein expression levels of p‐PI3K/PI3K, p‐AKT/AKT, p‐mTOR/mTOR, RAS and p‐ERK1/2/ERK1/2 showed obvious increase (*p* < 0.01) after the addition of Circ‐9110. Comparing with the pc2.1‐Circ‐9110 group, the Circ‐9110+miR‐100‐5p group reduced the protein levels of p‐PI3K/PI3K, p‐AKT/AKT, p‐mTOR/mTOR, RAS and p‐ERK1/2/ERK1/2 (Figure 5C, *p* < 0.01). Circ‐9110 to siRNA1 and siRNA2 knocked down the protein levels of p‐PI3K/PI3K, p‐AKT/AKT, p‐mTOR/mTOR, RAS and p‐ERK1/2/ERK1/2 (Figure 5D, *p* < 0.01).

### 3.9 MiR‐100‐5p reduced embryo implantation in vivo

**FIGURE 6:**
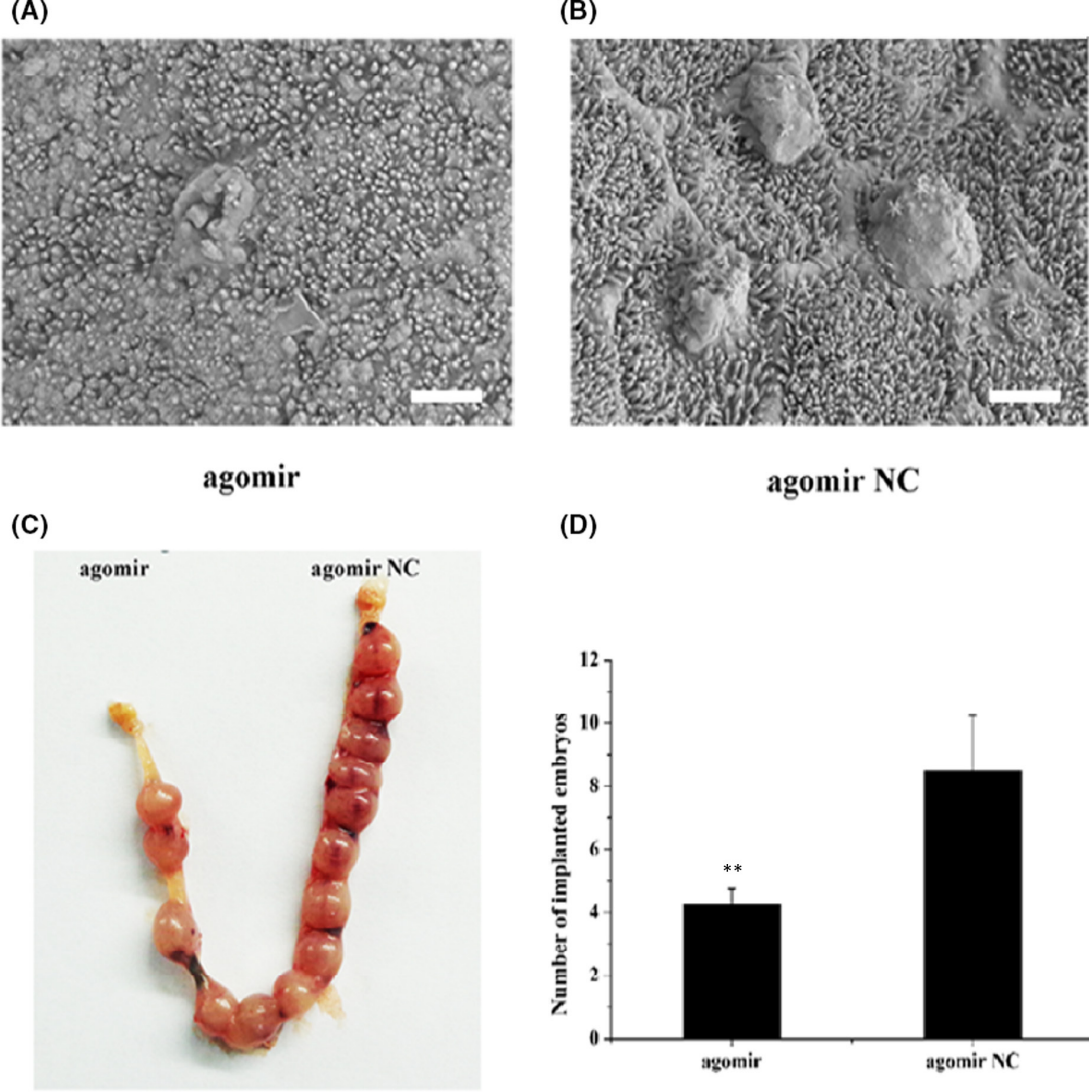
MiR‐100‐5p reduced embryo implantation in vivo. Note: Surface morphology of the endometrium under a scanning electron microscope. (A) Mice were injected with the miR‐100‐5p agomir. Scale bar: 2 μm (B) Mice were injected with the agomir NC. Scale bar: 2 μm (C) Mice uterus was injected with miR‐100‐5p agomir and agomir NC at day 3 of pregnancy, and, observed on day 9. (D) Number of embryo implantation was significantly decreased in the uterine horn injected with miR‐100‐5p agomir. The values are shown as mean ± SEM for three individuals. ** indicates that *p *< 0.01

We further verified the role of miR‐100‐5p in uterine receptivity and embryo implantation by mouse models in vivo. The SEM images showed that more rich pinopodes were observed on the endometrial surface in the agomir NC group compared with the miR‐100‐5p agomir group (Figure 6A,B). Expression patterns of *HOXA1* proteins in dairy goat endometrium was shown in Figure S7, which showed a higher expression in the agomir NC group. As seen in Figure 6C, miR‐100‐5p was injected into one horn of mouse uterus while NC was injected into the other horn as control at D3 of pregnancy. Meanwhile, in bar graph, it was clearly found that miR‐100‐5p significantly decreased embryo implantation rate in mouse dissected at D9 of pregnancy (Figure 6D).

## 4 DISCUSSION

In this study, the miR‐100‐5p levels significantly decreased in RE, consistent with the previous sequencing data. Caprine miRNAs in the RE and PE libraries showed that miR‐100‐5p which is one of the 33 down‐expressed miRNAs decreased 1.14‐fold in the RE relative to that in PE in dairy goat.^14^ The expression patterns and epigenetic regulation of miRNAs were found on the endometrium in the PE and RE.^13^, ^19^ Furthermore, some lncRNAs and miRNAs acted as regulators in endometrium cells and contributed to endometrial receptivity.^14^, ^20^ Therefore, miR‐100‐5p maybe exert critical role in regulation of endometrial receptivity.

The endometrium underwent a series of events including cell proliferation and cell apoptosis during the oestrous cycle and embryo implantation in many mammals.^21^, ^22^ Consistent with other special miRNAs, in this study, overexpressed miR‐100‐5p prevented ESCs proliferation and induced ESCs apoptosis. Contrarily, miR‐100‐5p inhibitor promoted ESCs proliferation and inhibited ESCs apoptosis.

In this study, it was confirmed that miR‐100‐5p directly targeted 3’UTR of *HOXA1* by luciferase assay as same as previous findings.^23^ And miR‐100‐5p decreased *HOXA1* mRNA and protein levels in caprine ESCs. *Hox* genes, which were related to miRNAs, acted as leading candidates in regulating the differentiation of the endometrium during embryonic implantation.^24^, ^25^ Based on the data that the *HOXA1* gene expression was upregulated in the RE compared with in the PE, overexpressed *HOXA1* stable ESCs cell line was established. The overexpression of *HOXA1* in ESCs significantly enhanced cell proliferation and reduced cell apoptosis compared with those in the control group. By contrast, the knockdown of *HOXA1* by siRNA suppressed cell proliferation and induced cell apoptosis in the ESCs. So, these results suggested that *HOXA1*, a miR‐100‐5p target gene, regulated ESCs proliferation and apoptosis.

In previous studies, it was reported that miR‐100‐5p inhibited the activity of mTOR and facilitated the autophagy of hepatocellular carcinoma cells.^26^ MiR‐100‐5p decreases cell proliferation by potentially downregulating the mTOR pathway and reduces MTOR protein levels after 72 h in LNCaP cells.^27^ Similarly, as shown in Figure S8, we found that the relative protein expression levels of p‐PI3K/PI3K, p‐AKT/AKT, p‐mTOR/mTOR and p‐ERK1/2/ERK1/2 on mTOR pathway showed substantially decrease (*p* < 0.01) after the miR‐100‐5p incubation. In addition, *HOXA1* promoted the phosphorylation level of PI3K, AKT and mTOR, indicating that *HOXA1* promoted cell proliferation and inhibited cell apoptosis via the PI3K/AKT/mTOR and ERK1/2 pathways. Cadherin‐6 and ephrin type‐A receptor 2(EphA2) are the direct downstream targets of *HOXA1*.^28^, ^29^ The overexpression of *HOXA1* inhibited apoptosis in human mammary carcinoma via activating E‐cadherin signalling pathway cells.^29^ Growth factor‐mediated EphA2 activated IRS1 and RAS by PI3K/AKT/mTOR pathway.^30^, ^31^ We speculate that, in our study, *HOXA1* may target EphA2 and CAD6, promote cell proliferation and decrease cell apoptosis in caprine ESCs through the PI3K/AKT/mTOR. Further research to confirm this hypothesis is highly desirable.

Luciferase assay showed that Circ‐9110 served as a sponge for miR‐100‐5p. CircRNAs usually acted as ceRNA binding miRNAs to regulate cell proliferation and apoptosis in various cells.^32^ Our study also verified that overexpressed circ‐9110 promoted ESCs proliferation and inhibited ESCs apoptosis, while the knockdown of circ‐9110 induced ESCs apoptosis and prevented ESCs proliferation. The relative protein expression levels of p‐PI3K/PI3K, p‐AKT/AKT, p‐mTOR/mTOR, RAS and p‐ERK1/2/ERK1/2 showed substantially increase after the introduction of Circ‐9110 in ESCs in vitro.

Pinopodes seem to be a useful maker for endometrium receptivity in many species.^33^, ^34^ In this study, the SEM observation showed that the number of pinopodes was significantly different between agomir NC group and miR‐100‐5p agomir group. The number of pinopodes and length of microvilli in miR‐100‐5p agomir group significantly decreased compared with agomir NC group. So, it was clearly showed that miR‐100‐5p may participate in the regulation of endometrial receptivity. Besides, the mouse model showed that miR‐100‐5p expression levels in vivo resulted in low embryo implantation rate. In disease association studies, miRNAs were involved in the process of uterine receptivity and embryo implantation.^35^, ^36^ Thus, this alteration in expression of pinopodes results in an alteration in the window of implantation. In the present study, we explored the effects of the miR‐100‐5p on endometrial receptivity and embryo implantation for the first time.

## 5 CONCLUSION

Taken together, we confirmed the role of miR‐100‐5p on endometrial receptivity and embryo implantation. Circ‐9110/miR‐100‐5p/*HOXA1* axis can promote ESCs proliferation and prevented ESCs apoptosis in vitro by linking PI3K/AKT/mTOR and ERK1/2 pathways. Besides, a sustained high level of miR‐100‐5p is detrimental to embryo implantation. Downregulation of miR‐100‐5p could thus be a biomarker to improve the endometrial receptivity function in dairy goats.

## CONFLICTS OF INTEREST

The authors declare that they have no conflicts of interest with the contents of this article.

## AUTHOR CONTRIBUTIONS

**Li Ma:** Conceptualization (lead); Writing – original draft (lead). **Meng Zhang:** Conceptualization (equal). **Fangjun Cao:** Conceptualization (equal); Data curation (equal); Writing – review & editing (equal). **Jincheng Han:** Conceptualization (supporting). **Peng Han:** Writing – review & editing (supporting). **Yeting Wu:** Investigation (equal). **Renyi Deng:** Writing – review & editing (equal). **Guanghui Zhang:** Data curation (supporting). **Xiaopeng An:** Formal analysis (supporting). **Lei Zhang:** Resources (equal). **Yuxuan Song:** Supervision (equal). **Binyun Cao:** Conceptualization (lead); Funding acquisition (lead).

## Supporting information

Supplementary Material

## Data Availability

All data generated or analysed during this study are included in this published article.

## References

[1] Morelli SS , Yi P , Goldsmith LT , et al. Endometrial stem cells and reproduction. Obstet Gynecol Int. 2012;2012:1‐5. doi:10.1155/2012/851367 PMC3263645 22287970

[2] Evron A , Goldman S , Shalev E , et al. Effect of primary human endometrial stromal cells on epithelial cell receptivity and protein expression is dependent on menstrual cycle stage. Hum Reprod. 2010;26:176‐190. 21098625 10.1093/humrep/deq296

[3] Zhang S , Kong S , Lu J , et al. Deciphering the molecular basis of uterine receptivity. Mol Reprod Dev. 2013;80:8‐21. 23070972 10.1002/mrd.22118

[4] Von Grothusen C , Lalitkumar S , Rao Boggavarapu N , et al. Recent advances in understanding endometrial receptivity: molecular basis and clinical applications. Am J Reprod Immunol. 2014;72:148‐157. 24635108 10.1111/aji.12226

[5] Zhang L , An XP , Liu XR , et al. Characterization of the transcriptional complexity of the receptive and pre‐receptive endometria of dairy goats. Sci Rep. 2015;5:14244. 26373443 10.1038/srep14244 PMC4571617

[6] Kim HW , Haider HK , Jiang S , et al. Ischemic preconditioning augments survival of stem cells via miR‐210 expression by targeting caspase‐8‐associated protein 2. J Biol Chem. 2009;284:33161‐33168. 19721136 10.1074/jbc.M109.020925 PMC2785158

[7] Mansfield JH , Harfe BD , Nissen R , et al. MicroRNA‐responsive'sensor'transgenes uncover Hox‐like and other developmentally regulated patterns of vertebrate microRNA expression. Nat Genet. 2004;36:1079‐1083. 15361871 10.1038/ng1421

[8] Subramanian S , Steer CJ . MicroRNAs as gatekeepers of apoptosis. J Cell Physiol. 2010;223:289‐298. 20112282 10.1002/jcp.22066

[9] Ren L , Chen W , Li S , et al. MicroRNA‐183 promotes proliferation and invasion in oesophageal squamous cell carcinoma by targeting programmed cell death 4. Br J Cancer. 2014;111:2003. 25211657 10.1038/bjc.2014.485 PMC4229630

[10] An XP , Ma HD , Liu YH , et al. Effects of miR‐101‐3P on goat granulosa cells in vitro and ovarian development in vivo via STC1. J Anim Sci Biotechnol. 2020;11:102. 33072314 10.1186/s40104-020-00506-6 PMC7557009

[11] Zhang Y , Liu JD , Li WF , et al. A regulated circuit orchestrated by novel‐miR‐3880 modulates mammary gland development. Front Cell Dev Biol. 2020;8:00383. 10.3389/fcell.2020.00383 PMC7325939 32656203

[12] Liu XR , Zhang L , Liu YX , et al. circ‐8073 regulates CEP55 by sponging miR‐449a to promote caprine endometrial epithelial cells proliferation via the PI3K/AKT/mTOR pathway. Mol Cell Res. 2018;1865:1130‐1147. 10.1016/j.bbamcr.2018.05.011 29800603

[13] An X , Liu X , Zhang L , et al. MiR‐449a regulates caprine endometrial stromal cell apoptosis and endometrial receptivity. Sci Rep. 2017;7:12248. 28947781 10.1038/s41598-017-12451-y PMC5612931

[14] Song Y , An X , Zhang L , et al. Identification and profiling of microRNAs in goat endometrium during embryo implantation. PLoS One. 2015;10:e0122202. 25886011 10.1371/journal.pone.0122202 PMC4401794

[15] Zhang L , Liu X , Liu J , et al. miR‐26a promoted endometrial epithelium cells (EECs) proliferation and induced stromal cells (ESCs) apoptosis via the PTEN‐PI3K/AKT pathway in dairy goats. J Cell Physiol. 2018;233:4688‐4706. 29115668 10.1002/jcp.26252

[16] Gu X , Li M , Jin Y , et al. Identification and integrated analysis of differentially expressed lncRNAs and circRNAs reveal the potential ceRNA networks during PDLSC osteogenic differentiation. BMC Genet. 2017;18:100. 29197342 10.1186/s12863-017-0569-4 PMC5712120

[17] Fu L , Jiang Z , Li T , et al. Circular RNA s in hepatocellular carcinoma: functions and implications. Cancer Med. 2018;7:3101‐3109. 10.1002/cam4.1574 PMC6051148 29856133

[18] Wei X , Li H , Yang J , et al. Circular RNA profiling reveals an abundant circLMO7 that regulates myoblasts differentiation and survival by sponging miR‐378a‐3p. Cell Death Dis. 2017;8:e3153. 29072698 10.1038/cddis.2017.541 PMC5680912

[19] Zhang L , Liu X , Ma X , et al. Testin was regulated by circRNA3175‐miR182 and inhibited endometrial epithelial cell apoptosis in prereceptive endometrium of dairy goats. J Cell Physiol. 2018;233:6965‐6974. 10.1002/jcp.26614 29693265

[20] Liu X , Zhang L , Liu Y , et al. Circ‐8073 regulates CEP55 by sponging miR‐449a to promote caprine endometrial epithelial cells proliferation via the PI3K/AKT/mTOR pathway. Biochim Biophys Acta Mol Cell Res. 2018;1865:1130‐1147. 29800603 10.1016/j.bbamcr.2018.05.011

[21] Wang Y , Xue S , Liu X , et al. Analyses of long non‐coding RNA and mRNA profiling using RNA sequencing during the pre‐implantation phases in pig endometrium. Sci Rep. 2016;6:20238. 26822553 10.1038/srep20238 PMC4731748

[22] Tranguch S , Dey SK . A lifetime of deciphering complexities of embryo implantation. Int J Dev Biol. 2014;58:79‐86. 25023673 10.1387/ijdb.130332st

[23] Zhang L , Liu X , Liu J , et al. miR‐182 aids in receptive endometrium development in dairy goats by down‐regulating PTN expression. PLoS One. 2017;12:e0179783. 28678802 10.1371/journal.pone.0179783 PMC5497977

[24] Xiao F , Bai Y , Chen Z , et al. Downregulation of *HOXA1* gene affects small cell lung cancer cell survival and chemoresistance under the regulation of miR‐100. Eur J Cancer. 2014;50:1541‐1554. 24559685 10.1016/j.ejca.2014.01.024

[25] Hoss AG , Kartha VK , Dong X , et al. MicroRNAs located in the Hox gene clusters are implicated in huntington's disease pathogenesis. PLoS Genet. 2014;10:e1004188. 24586208 10.1371/journal.pgen.1004188 PMC3937267

[26] Taylor HS . The role of HOX genes in human implantation. Hum Reprod Update. 2000;6:75‐79. 10711832 10.1093/humupd/6.1.75

[27] Ge YY , Shi Q , Zheng ZY , et al. MicroRNA‐100 promotes the autophagy of hepatocellular carcinoma cells by inhibiting the expression of mTOR and IGF‐1R. Oncotarget. 2014;5:6218. 25026290 10.18632/oncotarget.2189 PMC4171624

[28] Nabavi N , Saidy NRN , Venalainen E , et al. MiR‐100‐5p inhibition induces apoptosis in dormant prostate cancer cells and prevents the emergence of castration‐resistant prostate cancer. Sci Rep. 2017;7:4079. 28642484 10.1038/s41598-017-03731-8 PMC5481412

[29] Chen J , Ruley HE . An enhancer element in the EphA2 (Eck) gene sufficient for rhombomere‐specific expression is activated by *HOXA1* and *HOXB1* homeobox proteins. J Biol Chem. 1998;273:24670‐24675. 9733765 10.1074/jbc.273.38.24670

[30] Zhang X , Emerald BS , Mukhina S , et al. *HOXA1* is required for E‐cadherin‐dependent anchorage‐independent survival of human mammary carcinoma cells. J Biol Chem. 2006;281:6471‐6481. 16373333 10.1074/jbc.M512666200

[31] Carriere A , Romeo Y , Acosta‐Jaquez HA , et al. ERK1/2 phosphorylate Raptor to promote Ras‐dependent activation of mTOR complex 1 (mTORC1). J Biol Chem. 2011;286:567‐577. 21071439 10.1074/jbc.M110.159046 PMC3013016

[32] Cui XD , Lee MJ , Kim JH , et al. Activation of mammalian target of rapamycin complex 1 (mTORC1) and Raf/Pyk2 by growth factor‐mediated Eph receptor 2 (EphA2) is required for cholangiocarcinoma growth and metastasis. Hepatology. 2013;57:2248‐2260. 23315987 10.1002/hep.26253

[33] Liu T , Liu S , Yu X , et al. Circular RNA‐ZFR inhibited cell proliferation and promoted apoptosis in gastric Cancer by sponging miR‐130a/miR‐107 and modulating PTEN. Cancer Res Treat. 2018;50:1396. 29361817 10.4143/crt.2017.537 PMC6192924

[34] Dippolito S , Nicuolo FD , Papi M , et al. Expression of pinopodes in the endometrium from recurrent pregnancy loss women. Role of thrombomodulin and ezrin. J Clin Med. 2020;9:2634. 10.3390/jcm9082634 PMC7464296 32823767

[35] Quinn CE , Casper RF , et al. Pinopodes: a questionable role in endometrial receptivity. Hum Reprod Update. 2008;15:229‐236. 18997181 10.1093/humupd/dmn052

[36] Liang J , Cao D , Zhang X , et al. MiR‐192‐5p suppresses uterine receptivity formation through impeding epithelial transformation during embryo implantation. Theriogenology. 2020;157:360‐371. 32861000 10.1016/j.theriogenology.2020.08.009

