# Supplementary material for: Effect of MiR‐100‐5p on proliferation and apoptosis of goat endometrial stromal cell in vitro and embryo implantation in vivo
Source: J Cell Mol Med. 2022 Apr 12;26(9):2543–56. doi: 10.1111/jcmm.17226 (PMC9077292; doi:10.1111/jcmm.17226)
Supplement: Supplementary file 1 — Supplementary Material [file JCMM-26-2543-s001.docx]

Supporting Information

**The Effect of MiR-100-5p on Proliferation and Apoptosis of Goat Endometrial Stromal Cell *in Vitro* and Embryo Implantation *in Vivo***

*Li Ma^1,2,a^, Meng Zhang^1,a^, Fangjun Cao^1,3,a^, Jincheng Han^1^, Peng Han^1^, Yeting Wu^4^, Renyi Deng^5^, Guanghui Zhang^6^, Xiaopeng An^1^, Lei Zhang^1^, Yuxuan Song^1*^ and Binyun Cao^1*^*

^1^College of Animal Science and Technology, Northwest A&F University, Yangling, Shaanxi 712100, P.R. China;

^2^Shaanxi University of Chinese Medicine, Xianyang, Shaanxi 712046, P.R. China;

^3^Shaanxi Institute of Zoology, Xi’an, Shaanxi 710072, P. R. China

^4^College of Food Science and Engineering, Northwest A&F University, Yangling, Shaanxi 712100, P.R. China

^5^Department of Foreign Languages, Northwest A&F University, Yangling, Shaanxi 712100, P.R. China

^6^College of Innovation and Experiment, Northwest A&F University, Yangling, Shaanxi 712100, P.R. China

**
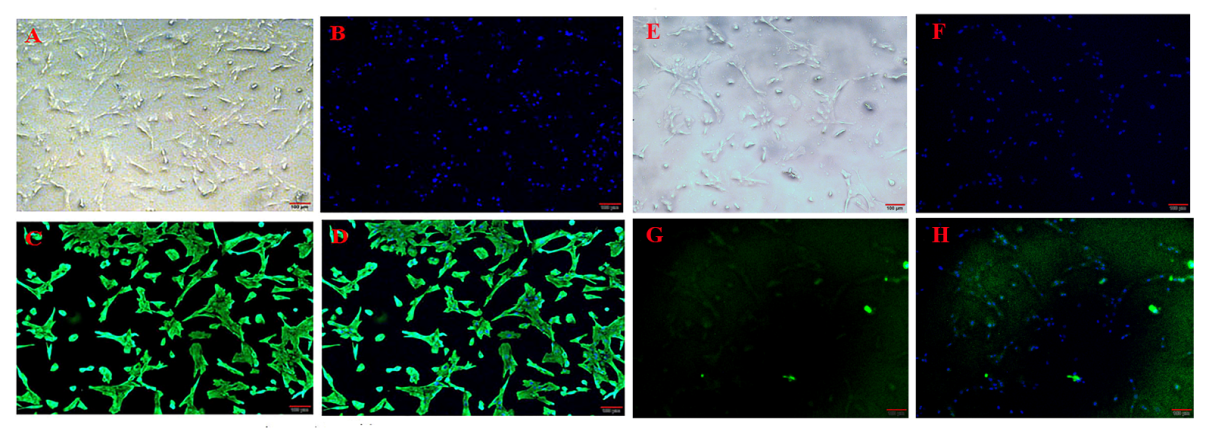
**

**Fig. S1.** Immunocytochemical analysis of vimentin and cytokeratin in endometrial stromal cell (ESCs). The fibre structure (A) and expression of vimentin (C) were shown in the primary ESCs. The cell nuclei were counterstained with DIPA (B). (D) showed the merged image. The fibre structure (E) and expression of cytokeratin (G) were shown in the primary ESCs. The cell nuclei were counterstained with DIPA (F), and (H) shows the merged image. Scale bars=100 µm.

**
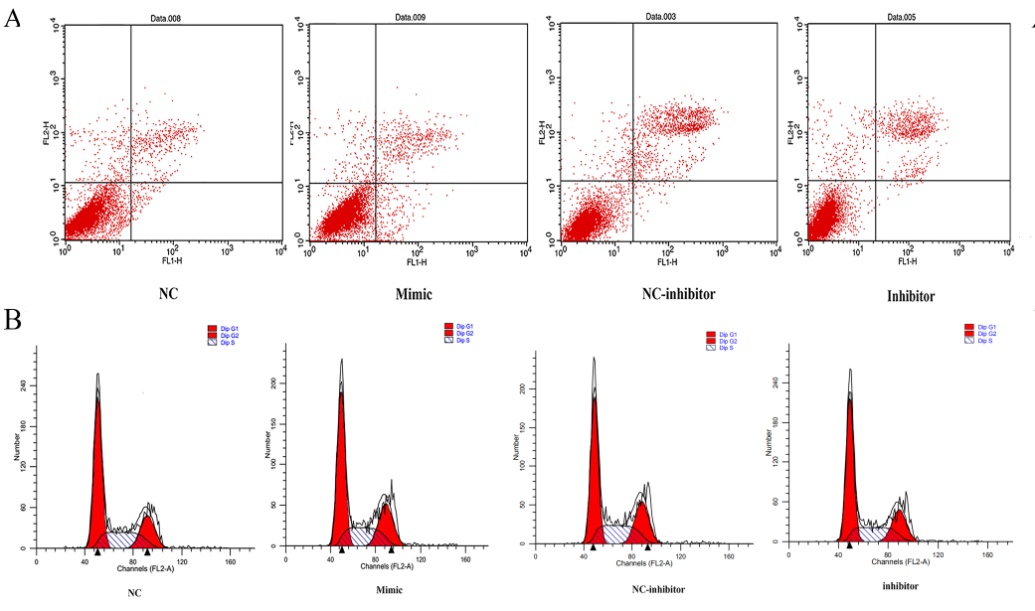
**

**Fig. S2.** MiR-100-5p induced ESCs apoptosis. Note: (A) ESCs were transfected with NC, miR-100-5p, NC-inhibitor or miR-100-5p inhibitor, and the apoptosis analysis of ESCs was performed with FCM. (B) ESCs were transfected with NC, miR-100-5p, NC-inhibitor or miR-100-5p inhibitor, and cell phases were analyzed by FCM.

**
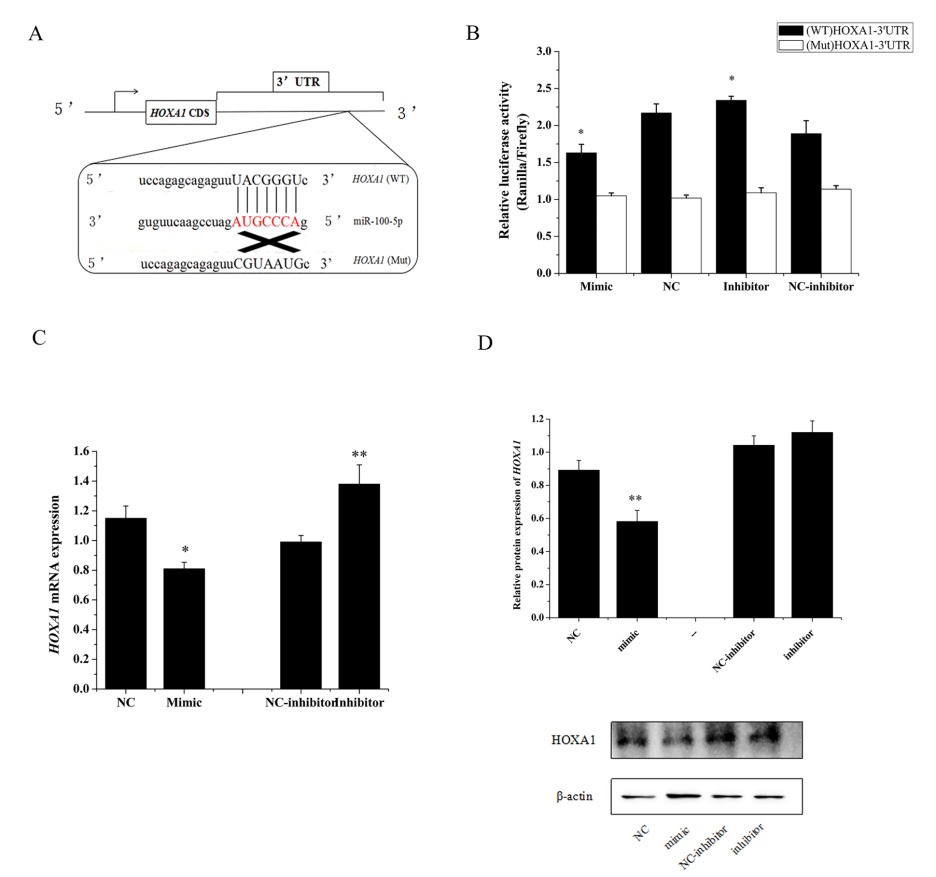
**

**Fig. S3.** *HOXA1* was a target gene of miR-100-5p. Note: (A) Schematic illustrating the design of luciferase reporters with WT-HOXA1 or a site-directed mutant (MUT-*HOXA1*). The nucleotides in red represent the ‘seed sequence’ of miR-100-5p. (B) The luciferase reporter assay of 293T cells co-transfected with WT-*HOXA1*-3′UTR or MUT-*HOXA1*-3′UTR and miR-100-5p mimic, NC, miR-100-5p inhibitor or NC-inhibitor. (C) miR-100-5p decreased *HOXA1* mRNA level in the ESCs. (D) miR-100-5p decreased *HOXA1* protein level in the ESCs. The values are shown as mean ± SEM for three individuals. ** indicates that *p*<0.01; * indicates that *p*<0.05.


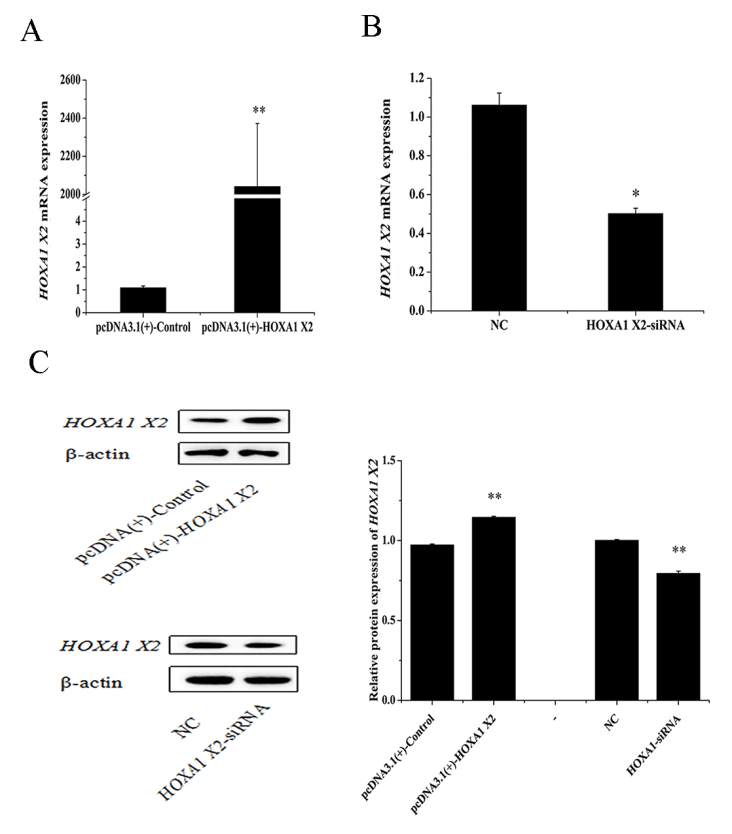


**Fig. S4.** The efficiency of *HOXA1* transfection in ESCs. (A) The levels of *HOXA1* in ESCs transfected with pcDNA3.1 or overexpressed *HOXA1* (pcDNA3.1(+)-HOXA1). (B) The efficiency of *HOXA1*-siRNA transfection in ESCs. (C) The protein levels of *HOXA1* after transfection with pcDNA3.1(+)-*HOXA1* or *HOXA1*-siRNA were measured by WB, and densitometry was normalized to the *β*-actin from the same lane. The values are shown as mean ± SEM for three individuals. ** indicates that *p*<0.01; * indicates that *p*<0.05.


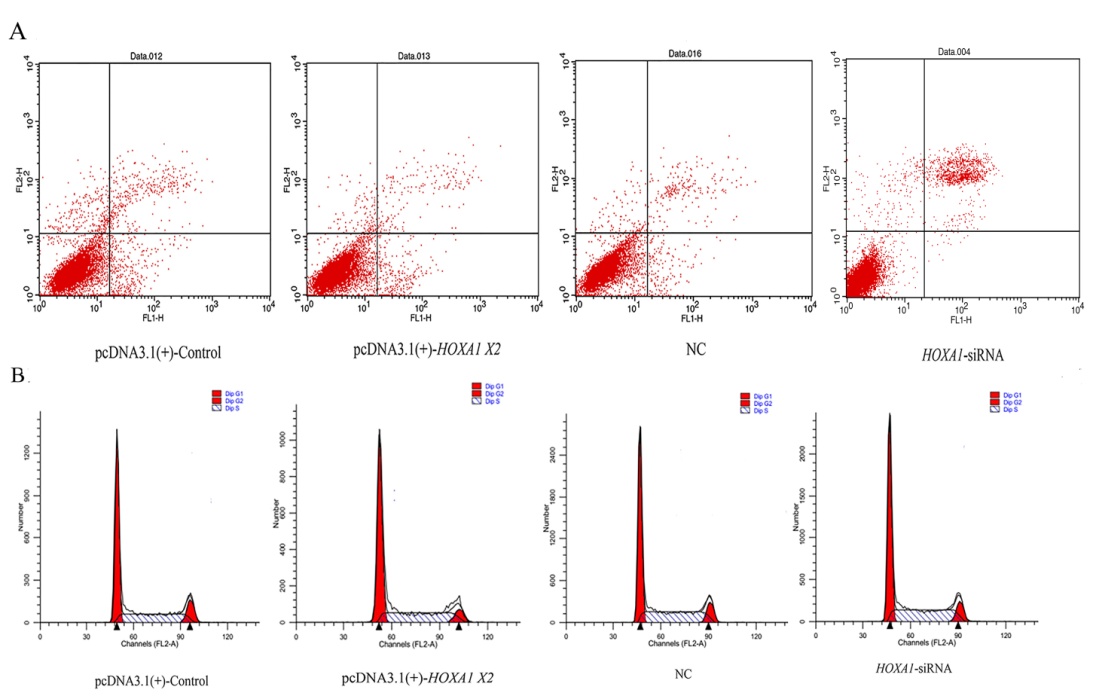


**Fig. S5.** *HOXA1* inhibited ESC apoptosis. Note: (A) ESCs were transfected with pcDNA3.1, pcDNA3.1(+)-*HOXA1*, NC or *HOXA1*-siRNA, and the apoptosis analysis of ESCs was performed with FCM. (B) ESCs were transfected with pcDNA3.1, pcDNA3.1(+)-*HOXA1*, NC or *HOXA1*-siRNA, and cell phases were analyzed by FCM.

**
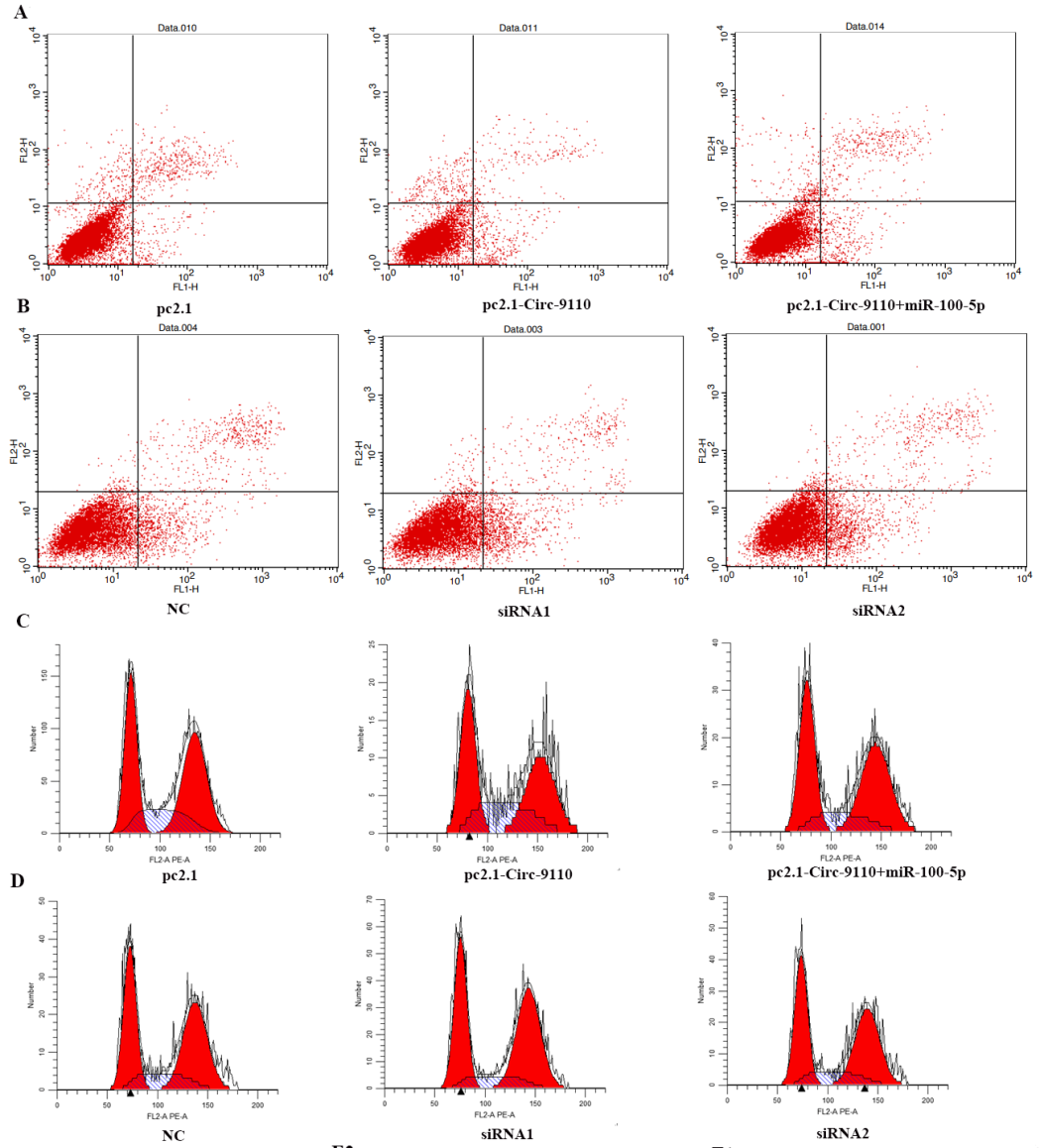
**

**Fig. S6.** Circ-9110 inhibited ESC apoptosis. Note: (A) ESCs were transfected with pc2.1, pc2.1-Circ-9110 or pc2.1-Circ-9110+miR-100-5p, and the apoptosis analysis of ESCs was performed with FCM. (B) ESCs were transfected with NC, siRNA1 or siRNA2, and the apoptosis analysis of ESCs was performed with FCM. (C) ESCs were transfected with pc2.1, pc2.1-Circ-9110 or pc2.1-Circ-9110+miR-100-5p, and cell phases were analyzed by FCM. (D) ESCs were transfected with NC, siRNA1 or siRNA2, and the cell phases were analyzed by FCM.

**Fig. S7.** Immunohistochemistry of mouse uterine horn for *HOXA1* protein in novel-miR-100-5p and agomir NC groups. Note: Immunohistochemistry for the *HOXA1* protein was performed in the uterine horn. LE=luminal epithelium, GE=glandular epithelium, SC= stroma cell, VE=vascular endothelial cell. Scale bars=50 μm, original magnification ×500.

**
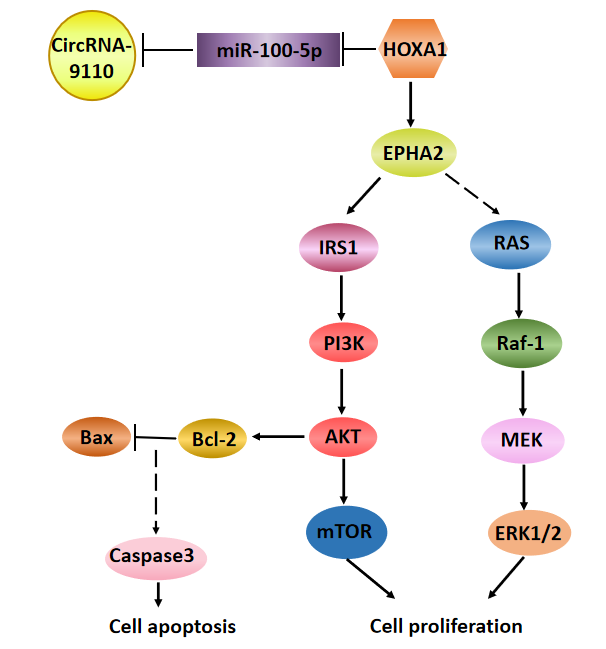
**

**Fig. S8.** Model of Circ-9110 regulatory mechanism on ESCs. In this model, Circ-9110 regulates ESCs by functioning as a ceRNA for miR-100-5p and shows a circRNA-miRNA-mRNA network thereby exerting biological effects.
